# Structural Analysis of the Hanks-Type Protein Kinase YabT From *Bacillus subtilis* Provides New Insights in its DNA-Dependent Activation

**DOI:** 10.3389/fmicb.2018.03014

**Published:** 2019-01-08

**Authors:** Lei Shi, Andrea Cavagnino, Jean-Luc Rabefiraisana, Noureddine Lazar, Inès Li de la Sierra-Gallay, Françoise Ochsenbein, Marie Valerio-Lepiniec, Agathe Urvoas, Philippe Minard, Ivan Mijakovic, Sylvie Nessler

**Affiliations:** ^1^ Division of Systems and Synthetic Biology, Department of Chemical and Biological Engineering, Chalmers University of Technology, Gothenburg, Sweden; ^2^ Institute of Integrative Biology of the Cell (I2BC), CEA, CNRS, Université Paris-Sud, Université Paris-Saclay, Gif-sur-Yvette, France; ^3^ Novo Nordisk Foundation Center for Biosustainability, Technical University of Denmark, Kongens Lyngby, Denmark

**Keywords:** autophosphorylation, dimerization, regulatory mechanism, crystallization chaperone, spore development

## Abstract

YabT is a serine/threonine kinase of the Hanks family from *Bacillus subtilis*, which lacks the canonical extracellular signal receptor domain but is anchored to the membrane through a C-terminal transmembrane helix. A previous study demonstrated that a basic juxtamembrane region corresponds to a DNA-binding motif essential for the activation of YabT trans-autophosphorylation. YabT is expressed during spore development and localizes to the asymmetric septum where it specifically phosphorylates essential proteins involved in genome maintenance, such as RecA, SsbA, and YabA. YabT has also been shown to phosphorylate proteins involved in protein synthesis, such as AbrB and Ef-Tu, suggesting a possible regulatory role in the progressive metabolic quiescence of the forespore. Finally, cross phosphorylations with other protein kinases implicate YabT in the regulation of numerous other cellular processes. Using an artificial protein scaffold as crystallization helper, we determined the first crystal structure of this DNA-dependent bacterial protein kinase. This allowed us to trap the active conformation of the kinase domain of YabT. Using NMR, we showed that the basic juxtamembrane region of YabT is disordered in the absence of DNA in solution, just like it is in the crystal, and that it is stabilized upon DNA binding. In comparison with its closest structural homolog, the mycobacterial kinase PknB allowed us to discuss the dimerization mode of YabT. Together with phosphorylation assays and DNA-binding experiments, this structural analysis helped us to gain new insights into the regulatory activation mechanism of YabT.

## Introduction

In bacteria, many physiological processes are regulated by protein phosphorylation cascades (Jers et al., 2008). The phosphoenolpyruvate (PEP):carbohydrate phosphotransferase system (PTS), regulating the import of sugars used as carbon sources by the bacteria, was one of the first identified bacterial signaling pathways (Postma et al., 1993). The phosphate from PEP is transferred on His or Cys residues of the phosphorelay proteins. The numerous two-component systems, which regulate gene expression in response to various environmental signals, rely on transmembrane histidine kinases (Hoch, 2000). Phosphorylation of Ser/Thr/Tyr residues, which was long thought to be exclusive to eukarya, has also been observed in all bacterial phyla, however with a quite uneven distribution among bacterial species (Deutscher and Saier, 2005; Wehenkel et al., 2008; Shi et al., 2014a). Tyrosine phosphorylation is mainly catalyzed by the idiosyncratic BY-kinases (Olivares-Illana et al., 2008), which are present only in bacteria, whereas Ser/Thr phosphorylation is mainly performed by eukaryotic-like Hanks-type kinases (Leonard et al., 1998; Stancik et al., 2018).

A comprehensive interactome study focused on the model organism *Bacillus subtilis* demonstrated a high degree of connectivity among all types of protein kinases and identified several cross-phosphorylation events (Shi et al., 2014c). This suggested that bacterial protein kinases could form signaling networks similar to the phosphorylation cascades found to regulate essential cellular processes like cancer development in eukarya (Brognard and Hunter, 2011).

In *B. subtilis*, only three Ser/Thr kinases of the Hanks family have been identified to date: PrkC, PrkD/YbdM, and YabT (Kobir et al., 2014). PrkD/YbdM is a soluble cytosolic protein, which has been shown to regulate the histidine kinase of the DegS/DegU two-component system involved in the control of the transition growth phase (Mader et al., 2002; Jers et al., 2011). PrkC is a transmembrane protein with an extracellular PASTA domain recognizing peptidoglycan fragments (Yeats et al., 2002), which has been shown to regulate several important biological processes such as morphogenesis (Foulquier et al., 2014), cell division (Pompeo et al., 2015), and dormancy (Pompeo et al., 2018). PrkC is homologous to the protein kinase PknB from *Mycobacterium tuberculosis*, which crystal structure of the catalytic domain has been one of the first characterized bacterial Hanks-type kinases (Ortiz-Lombardia et al., 2003; Young et al., 2003). YabT lacks an extracellular sensing domain, but it is linked to the membrane *via* a C-terminal transmembrane helix (TM). Interestingly, the basic juxtamembrane region of the protein has been shown to be involved in DNA-dependent activation of YabT (Bidnenko et al., 2013). The same study showed that YabT is specifically expressed in sporulation and localizes to the asymmetric septum where it specifically phosphorylates the general DNA-recombinase RecA (Lusetti and Cox, 2002), thus ensuring chromosome integrity during spore development. In addition, YabT also phosphorylates the single-stranded DNA-binding protein SsbA (Derouiche et al., 2017) and YabA, the initiation-control protein, which is also involved in sporulation (Garcia Garcia et al., 2018), thus suggesting a central regulatory role for YabT in genome maintenance during spore development. YabT has also been shown to regulate protein synthesis through phosphorylation of AbrB, a global gene regulator involved in transition phase regulation (Kobir et al., 2014) and the universal elongation factor Ef-Tu (Pereira et al., 2015). Finally, YabT was also shown to phosphorylate other protein kinases such as the histidine kinase DegS, already mentioned above as substrate of PrkD/YbdM, the serine kinases RsbW and SpoIIAB from the histidine-kinase/HSP90-like ATPase superfamily, which regulate sigma-B and sigma-F activities, respectively (Shu et al., 2004; van Schaik et al., 2005), and the BY-kinase PtkA involved in several processes, including extracellular polysaccharide production, sporulation, and biofilm formation (Derouiche et al., 2013, 2015, 2016; Gerwig et al., 2014). Reciprocally, PtkA has been shown to phosphorylate YabT (Shi et al., 2014c).

Here, we determined the crystal structure of the bacterial DNA-dependent protein kinase YabT at 1.6 Å resolution. An original approach using a fusion with an artificial protein scaffold as crystallization helper was developed. Using NMR interaction experiments, we demonstrated that the juxtamembrane domain of YabT, which is disordered in the absence of DNA, is folded in the presence of DNA. In comparison with other bacterial Hanks-type kinases, in particular PknB, provided insights into the activation mechanism of YabT.

## Materials and Methods

### Protein Production and Purification

The gene coding for a truncated form of YabT, in which the putative C-terminal transmembrane (TM) helix (residues 316–338) was removed, has been cloned in QIAexpress pQE vector with either a N-terminal or a C-terminal 6xHis-tag. The recombinant proteins 6xHis-YabT(∆TM) and YabT(∆TM)-6xHis were produced in *Escherichia coli* M15 pRep4 and purified as described (Bidnenko et al., 2013) using Immobilized Metal Affinity Chromatography (IMAC). The lysis buffer was supplemented with 2 M NaCl in order to avoid residual binding of DNA. The purification protocol was completed by a Size Exclusion Chromatography (SEC) step using a Superdex S75 column. The purified proteins were highly soluble and could be concentrated up to 125 mg/ml.

A large library of 10^9^ artificial protein scaffolds called αREPs has been screened using phage display to select αREP variants displaying high affinity for YabT(∆TM) (Chevrel et al., 2017). The YabT-specific αREP binder called bE8 has been produced with a N-terminal 6xHis-tag and purified using IMAC.

Short and long forms of bE8-YabT(∆TM) fusion protein were constructed by inserting a short linker of 10 residues (SGGGGSGGGG) or a long linker of 32 residues (GSAGSAAGSGGASGGGGSGGGGSAGSAAGSGG) connecting 6xHis-bE8 to the N-terminus YabT(∆TM) (residues 1–315) (Chevrel et al., 2017).

Both fusion proteins were produced in *E. coli* BL21-Gold(DE3) and were purified using IMAC and a Superdex S200 SEC column.

The purity of the samples was checked by SDS-Polyacrylamide Gel Electrophoresis (SDS–PAGE). The purified proteins were conserved in 20 mM Tris-HCl (pH 7.5), 200 mM NaCl, and 5 mM β-mercaptoethanol.

### Oligomerization State Analysis

Size-exclusion chromatography-multi-angle light scattering (SEC-MALS) experiments were performed using a Viscotek TDA305 triple detector array with an integrated GPC_max_ VE 2001 system (Malvern, France). The fusion proteins were injected on a Agilent Bio Sec 3 column equilibrated in 20 mM Tris-HCl (pH 7.5), 200 mM NaCl, and 5 mM β-mercaptoethanol. The OmniSEC software of the manufacturer was used for acquisition and analysis of the data. Bovine Serum Albumin (BSA, Sigma–Aldrich) was used as standard reference protein for detector calibration.

### DNA-Binding Analysis

Electrophoretic mobility shift assays (EMSA) were performed as described previously (Bidnenko et al., 2013). Proteins were incubated with 90 nucleotide-long ssDNA or 210 bp-long dsDNA in the reactions containing 25 mM Tris-HCl (pH 7.5), 50 mM NaCl, 5% glycerol, 1 mM DTT, 10 mM MgCl_2_, 50 mg/ml BSA, and 1 mM ATP. 90 ssDNA was a random sequence oligo, and 210 bp dsDNA was the PCR product amplified from *B. subtilis* genomic DNA by primers NCterF and NCterR (Mijakovic et al., 2003). Reactions were incubated at 310 K for 30 min and analyzed by electrophoresis in 1.0% agarose gels. The gel was stained by GelRed Nucleic Acid Gel Stain (Biotium).

### *In vitro* Protein Phosphorylation Assays

*In vitro* phosphorylation assays were performed as described previously (Mijakovic et al., 2003). The protein samples were incubated with dsDNA in the presence of 25 mM Tris-HCl (pH 7.5), 50 mM NaCl, 5% glycerol, 1 mM DTT, 10 mM MgCl_2_, and 50 mg/ml BSA. The reactions were started by adding 10 mM ATP containing 20 mCi mmol^−1^ [γ-^32^P]-ATP. Radioactive phosphorylated proteins were revealed by autoradiography of SDS-PAGE using a phosphoimager from FUJI.

### Crystallization Assays

Crystallization trials were performed at 290 K using a Cartesian robot and commercial kits. Initial hits were reproduced and optimized manually using the hanging drop method and homemade solutions. The short form of bE8-YabT(∆TM) was crystallized in 5% PEG 8000 (w/v), 1 mM spermine, 20 mM MgCl_2_, and 0.05 M Hepes (pH 7.5) at a concentration of 6 mg/ml. The long form of the fusion protein was crystallized in 10% PEG 8000 (w/v), 8% (v/v) ethylene glycol, and 0.1 M Hepes (pH 7.5) at a concentration of 20 mg/ml. Crystals were flash frozen in the crystallization solution supplemented with 35% glycerol and conserved in liquid nitrogen prior X-ray diffraction assays.

### X-Ray Diffraction Data Collection and Processing

Crystals of the short and long forms of the bE8-YabT(∆TM) fusion protein diffracted up to 1.6 Å on beamline Proxima-1 at SOLEIL (Saint-Aubin, France) and 1.45 Å resolution on beamline ID30B at the European Synchrotron Radiation Facility (Grenoble, France), respectively. The diffraction data were processed using XDS (Kabsch, 2010), and the crystallographic phase problem was solved by molecular replacement using PHASER (McCoy et al., 2007). A YabT search model was calculated by homology modeling using the Fold and Function Assignment Server FFAS (Jaroszewski et al., 2005). Several structures of eukaryotic protein kinases displaying up to 30% sequence identity with YabT were used to create the model. The N-terminal region (residues 18–130) of an αREP binder containing 4 repeats (Protein Data Bank identifier (PDB ID): 3LTJ) (Urvoas et al., 2010) was used as template for the bE8 part of the fusion protein. The resulting model of the fusion protein was refined using PHENIX (Adams et al., 2010) and manually optimized using COOT (Emsley and Cowtan, 2004).

### NMR Analysis

Purified 6His-YabT(∆TM) was analyzed on a high-field NMR spectrometer (600 MHz, Bruker Avance III with TCI (^1^H, ^13^C, ^15^N, ^2^H) 5 mm, z-gradients cryoprobe). The uniformly ^15^N labeled protein was suspended at a concentration of 25 μM in 20 mM MES (pH 6.5), 100 mM NaCl, 4 mM MgCl_2_, and 8% D_2_O. ATP was added at a final concentration of 1 mM and a 25-mer forked DNA oligonucleotide at a final concentration of 4 mM. Sofast-HMQC experiments were recorded at 293 K with 512 scans and 256 experiments in the indirect dimension.

### Structure and Sequence Analysis

We used the protein structure comparison service PDBeFold^1^ (Krissinel and Henrick, 2004) and the Protein Interfaces, Surfaces, and Assemblies service PDBePISA^2^ (Krissinel and Henrick, 2007) at the European Bioinformatics Institute to compare crystal structures and analyze their interaction surfaces, respectively. We used the PyMOL Molecular Graphics System (DeLano, 2002) to analyze the 3D structures and prepare the figures of 3D structures. We used ESPript (Gouet et al., 2003) to prepare the sequence alignment with the secondary structure elements displayed on the top.

## Results

### Crystallization of YabT in Fusion With an αREP Binder

YabT(∆TM)-6xHis crystallized in several conditions, but the crystals only diffracted at low resolution and could not be used to solve the 3D structure of YabT(∆TM). The second construct 6xHis-YabT(∆TM) did not give better results, suggesting that the intrinsic flexibility of the protein was responsible for the low diffraction power of our crystals.

In order to stabilize YabT(∆TM) in a homogenous conformation, we decided to use artificial protein scaffolds called αREPs, which specifically bind to a protein target and have been shown to be useful as crystallization helpers (Valerio-Lepiniec et al., 2015). The structure of REPs is based on the thermostable HEAT-like repeat proteins (Urvoas et al., 2010). It displays a variable number of α-helical repeats carrying five highly randomized amino acid positions, which form a hypervariable surface ensuring specific recognition of the target protein (Guellouz et al., 2013). The selected YabT(∆TM)-specific αREP binder called bE8 is composed of two internal repeats framed by additional N- and C-Cap α-helical fragments (Chevrel et al., 2017).

By mixing purified samples of αREP and kinase in a 1:1 molar ratio, a stable bE8/YabT(∆TM) complex of about 50 kDa has been purified using size-exclusion chromatography (data not shown) and used to setup crystallization assays. Crystals diffracting up to 3.0 Å resolution were obtained, but they contained only the αREP binder bE8. To stabilize the bE8-YabT interaction, we decided to create a fusion protein.

**Figure 1:**
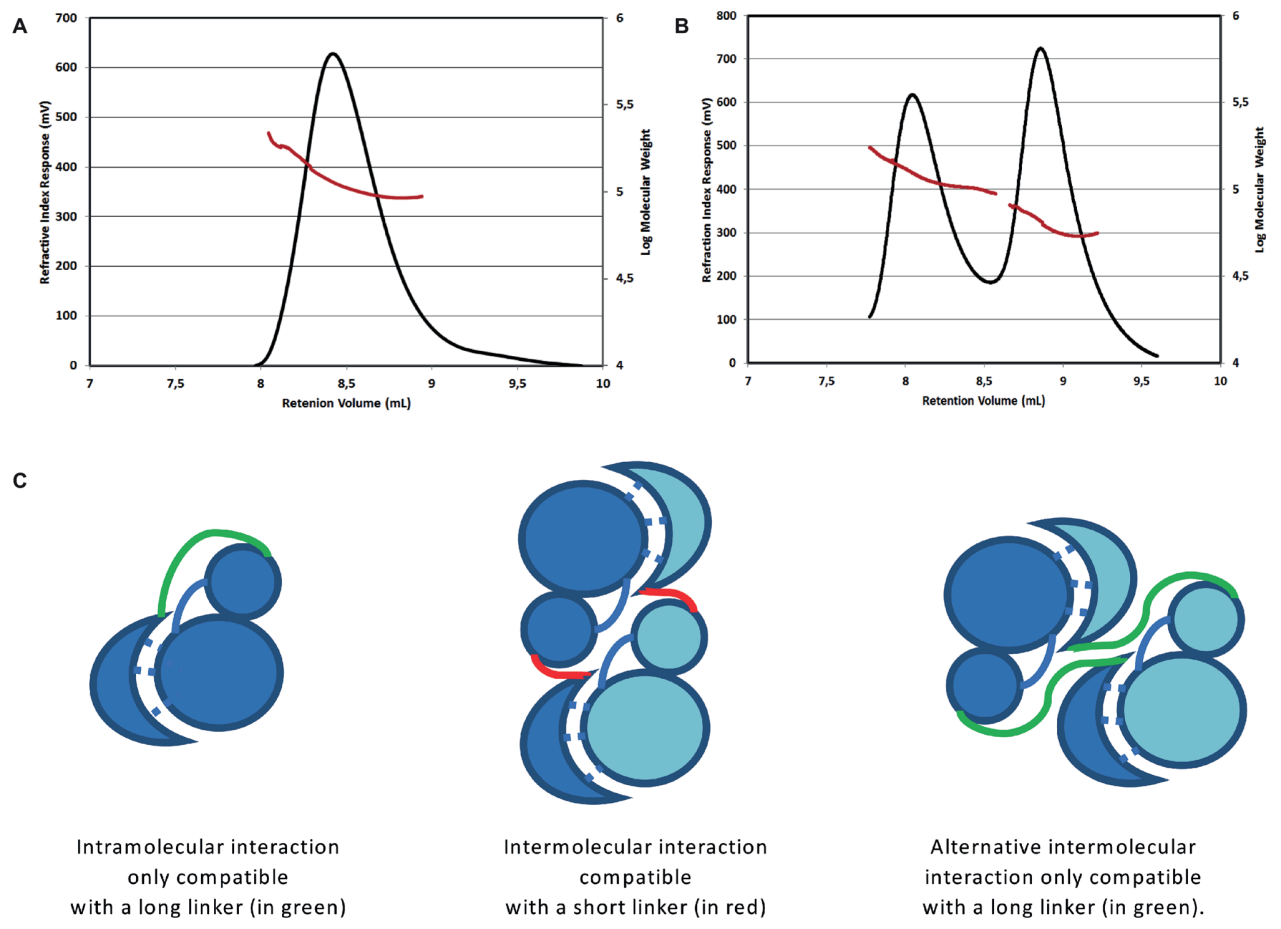
Oligomerization state of the short and long forms of the bE8-YabT(∆TM) fusion protein. SEC-MALS analysis of the short form injected at 3 mg/ml **(A)** and of the long form injected at 4 mg/ml **(B)**. The short form was eluted as a single peak corresponding to a dimer of about 115 kDa. The long form was eluted as two main peaks, which were analyzed as an equilibrium between a monomer of about 60 kDa and a dimer of about 120 kDa. An additional small peak suggested the formation of a tetramer. The two main peaks were analyzed separately but are displayed on the same graph. The elution profiles are represented according to retention volume (in ml) with the refractive index (in mV) indicated on the left axis (black curve) and the logarithm of molecular weight on the right axis (red curve). **(C)** Models of the intra- and inter-molecular interactions of the bE8 and YabT(∆TM) domains of the fusion proteins. The linker between bE8 and the bilobal kinase domain is shown in red for the short form and in green for the long form. The specific contacts between bE8 and YabT are shown as dashed lines. A subunit is colored in dark blue and the other one in pale blue.

Two bE8-YabT(∆TM) constructs were prepared: a short form with a linker of 10 residues and a long form with a linker of 32 residues. Interestingly, SEC-MALS analyses showed that the short version of the fusion protein (51 kDa) formed a stable dimer (Figure 1A), whereas the long fusion (53 kDa) was in equilibrium between monomer and dimer (Figure 1B). These results suggested that the short linker impairs the formation of an intramolecular interaction between the bE8 and YabT domains of the fusion protein, thus favoring the formation of intermolecular interactions through dimerization of the fusion protein (Figure 1C). The monomeric and dimeric forms observed with the long linker could in turn correspond to intra- and intermolecular interactions between the bE8 and YabT(∆TM) domains, respectively. The peak corresponding to the dimeric form of the long fusion protein was eluted earlier than the dimeric short form, suggesting an alternative dimerization mode (Figure 1C).

**Figure 2:**
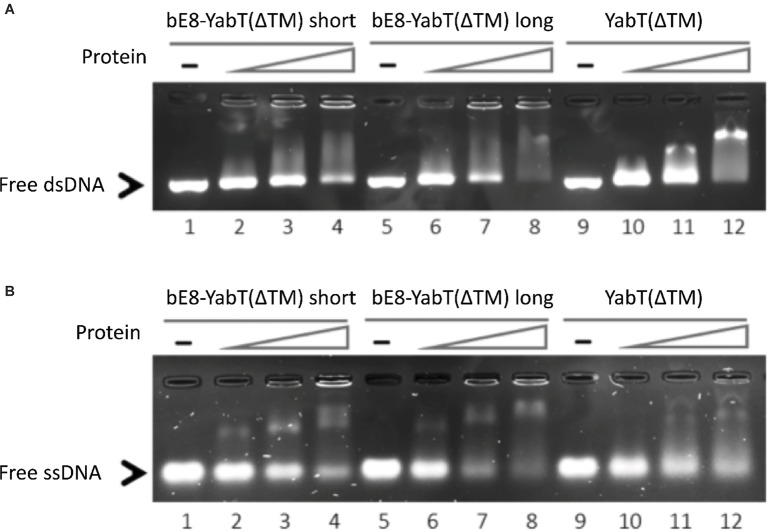
DNA-binding activity of the short and long forms of the bE8-YabT(∆TM) fusion protein. 0.075 μM dsDNA (210 bp) **(A)** and 0.15 μM ssDNA (90 nt) **(B)** were incubated at 310 K for 30 min with 0 μM (lane 1, 5 and 9), 0.4 μM (lane 2, 6 and 10), 0.8 μM (lane 3, 7 and 11), and 1.2 μM (lane 4, 8 and 12) of protein, respectively. The samples were then submitted to electrophoretic mobility shift assays (EMSA). The activity of YabT(∆TM) is used as positive control.

**Figure 3:**
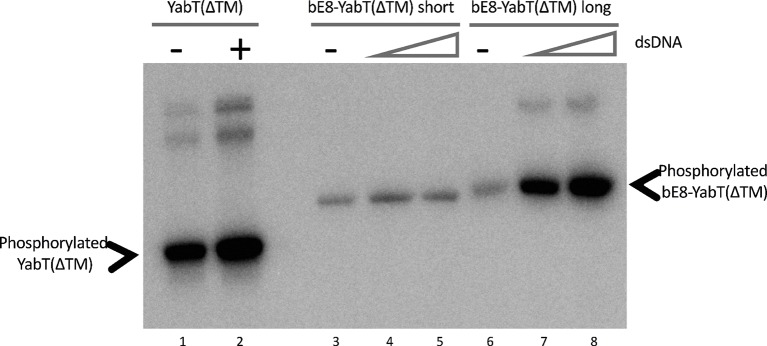
Autophosphorylation activity of the short and long forms of the bE8-YabT(∆TM) fusion protein. About 1.6 μM protein samples were incubated with 0 nM (lane 1, 3 and 6), 33 nM (lanes 2, 4, and 7), and 67 nM (lanes 5 and 8) dsDNA. Autophosphorylation reactions were started by adding 10 mM ATP containing 20 mCi mmol-1 [γ-^32^P]-ATP. The samples were analyzed by SDS-PAGE, and the radioactive phosphorylated proteins were revealed by autoradiography. The activity of the short and long forms of the bE8-YabT(∆TM) fusion protein is compared to the activity of a YabT(∆TM) sample used as positive control. Phosphorylated YabT and bE8-YabT samples are indicated by arrows. Upper band(s) are observed when YabT is active. They could correspond either to minor multimeric states of the phosphorylated protein or to a minor *E. coli* contaminant recognized as substrate by the poorly specific YabT.

EMSA experiments (Figure 2) demonstrated that both versions of the bE8-YabT(∆TM) fusion protein were able to bind ssDNA and dsDNA, with the long form being slightly more efficient. We also tested the effect of DNA binding on the activity of the fusion proteins. Because both auto- and substrate-phosphorylation processes rely on the same ATP-dependent mechanism, we only tested the trans-autophosphorylation activity of the fusion proteins (Figure 3). These assays showed that both forms of the fusion protein are poorly phosphorylated in the absence of DNA compared to YabT(∆TM). Furthermore, only the long form of the fusion protein was activated upon addition of DNA, suggesting that the dimeric short form of the fusion (Figure 1C) is incompatible with the DNA-dependent trans-autophosphorylation mechanism of YabT. Trans-autophosphorylation most probably requires the formation of a YabT dimer incompatible with the dimerization mode of the short bE8-YabT(∆TM) fusion protein. The more flexible long form of the fusion protein seems however to allow the formation of this active YabT dimer. It is also most probably compatible with substrate binding.

### Overall Structure of the αREP-YabT Fusion Protein

Crystallization assays were performed with the short form as well as with the long form of the bE8-YabT(∆TM) fusion protein. Both crystallized in similar conditions and yielded identical crystals diffracting at high resolution in space group P2_1_2_1_2, with one molecule of YabT(∆TM) and one bE8 binder in the asymmetric unit. Initial models of the kinase and αREP moieties were produced using homology modeling, and phasing was performed using molecular replacement. The structure refined against the 1.6 Å resolution diffraction data set obtained with a crystal of the short form of the fusion protein was deposited in the Protein Data Bank (PDB) under the accession code 6G4J. The statistics for data processing and model refinement are given in Table 1.

**Table 1:** X-ray diffraction data processing and refinement statistics.

Data processing statistics	bE8-YabT(∆TM)
Space group	P2_1_ 2_1_ 2
Unit cell parameters (Å)	*a* = 67.7; *b*=122.9; *c* = 50.6
Unit cell angles (°)	*α* = 90.0; *β* = 90.0; *γ* = 90.0
Resolution range (Å)*	50.0–1.6 (1.7–1.6)
No. of unique reflections	56 285 (8 958)
Completeness (%)	99.4 (98.8)
CC(1/2)	99.9 (53.2)
Mean I/σ(I)	14.11 (1.28)
R_meas_(%)^a^	5.3 (105.8)
**Refinement statistics**	
Resolution range	40.5–1.6 (1.63–1.60)
No. of molecules/a.u.	1
R_work_(%)^b^	18.89 (28.65)
R_free_(%)^c^	21.69 (31.44)
Ramachandran	
Favored (%)	99.2
Outliers (%)	0.0
R.M.S.D.	
Bond lengths (Å)	0.006
Bond angles (°)	1.017
Chirality	0.037
Planarity	0.005
Dihedral	11.916
Average B, all atoms (Å^2^)	32.66

* Numbers in parentheses represent values in the highest resolution shell.

a R_meas_ = ∑hkl [N/N−1]^1/2^∑i |Ii(hkl)−<I(hkl)>|/∑hkl∑i Ii(hkl), where N is the multiplicity of a given reflection, Ii(hkl) is the integrated intensity of a given reflection, and <I(hkl)> is the mean intensity of multiple corresponding symmetry-related reflections.

b R_work_ = ∑ ||Fobs| − |Fcalc||/∑ |Fobs|, where |Fobs| and |Fcalc| are the observed and calculated structure factor amplitudes, respectively.

c R_free_ is the same as R_work_ but calculated with a 20% subset of all reflections that was never used in refinement.

**Figure 4:**
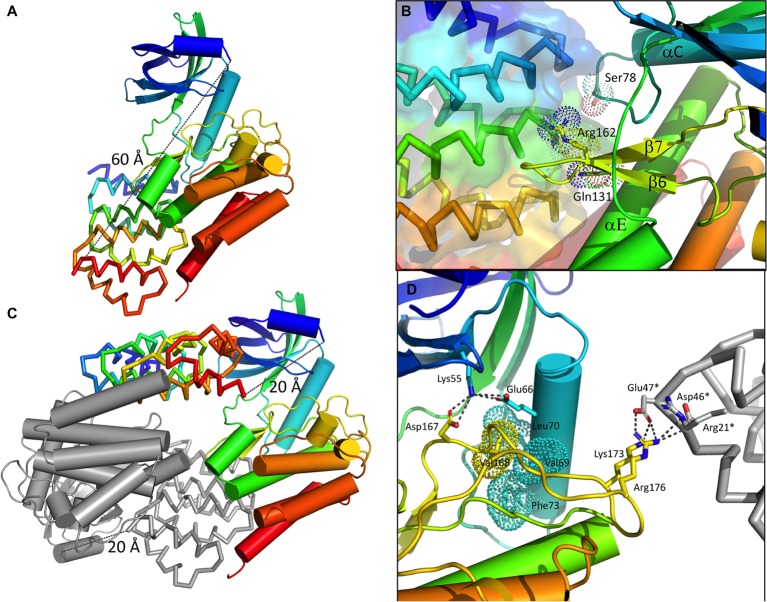
Structure of the bE8-YabT(∆TM) fusion proteins. **(A)** Overall structure of the bE8-YabT(∆TM) monomeric form. The YabT(∆TM) is shown in cartoon format and the bE8 αREP binder as ribbon. Both domains are colored in a rainbow scheme from blue (N-terminus) to red (C-terminus). The secondary structure elements of YabT(∆TM) are labeled. The linker region is not visible, but both domains display the characteristic *α*REP interaction mode. The distance of 60 Å separating the C-terminus of bE8 from the N-terminus of YabT is highlighted by a dashed line. It is compatible with a 32 residues long linker. **(B)** Close view of the bE8/YabT(∆TM) interaction. The bE8 surface is shown in transparency. YabT residues directly implicated in polar interactions with bE8 are labeled and shown in sticks and dots. **(C)** Alternative dimeric form of the bE8-YabT(∆TM) fusion protein observed in crystal packing. A subunit is shown in the same scheme as in panel A. The second subunit is shown in gray. In each subunit, the C-terminus of the bE8 domain is only 20 Å apart from the N-terminus of the YabT(∆TM) domain but specifically interact with the YabT(∆TM) domain of the second subunit. **(D)** Characteristics of the active closed conformation. The electrostatic interactions made by Lys55 from strand β3 and residues Lys173 and Arg176 from the activation loop are highlighted by dashed lines. The hydrophobic interactions between Val168 from the activation loop (yellow) and helix αC (cyan) are highlighted by dots. A neighboring bE8 molecule from the crystal packing is shown as gray ribbon with residues Arg21*, Asp46*, and Glu47* in sticks.

The YabT catalytic domain displays the classical bilobal architecture of Hanks-type kinases (Kornev and Taylor, 2010). The small N-lobe consists of a six-stranded antiparallel β-sheet (strands β0–β5) curled around an N-terminal helix called αB and packed against the regulatory helix called αC, following the classical nomenclature of Hanks-type kinases. The large C-lobe is composed of six α-helices (αD-αI), a 3_10_-helix (η1), and a β-hairpin (strands β6–β7). The nucleotide-binding site located in the cleft between the two lobes is empty (Figure 4A). The DNA-binding region (residues 274–315) is disordered and not visible in the electron density map.

The polypeptide linking the two domains of the fusion protein was not visible in the electron density map. The bE8 binder, which displays the classical αREP fold (Urvoas et al., 2010), interacts as expected with YabT(∆TM) through its concave face. Fifteen out of the eighteen variable residues of bE8 are involved in YabT(∆TM) binding (Chevrel et al., 2017). From the kinase point of view, the interaction mainly occurs through residues Arg162 from loop β6–β7, Gln131 from helix αE, and Ser78 from loop αC-β4 (Figure 4B). Structure analysis using the PDBePISA server (Krissinel and Henrick, 2007) confirmed that this interaction mode is stable in solution, with a buried area of 1710 Å^2^ and a solvation energy gain of −7.0 kcal/mol.

The distance between the C-terminus of bE8 and the N-terminus of YabT(∆TM) would be about 60Å long (Figure 4A). This distance is compatible with an extended conformation of the 32 residues long linker but not with its short version of 10 residues. Nevertheless, in the crystal packing, the C-terminus of each bE8 domain is only 20 Å apart from the N-terminus of a neighboring YabT(∆TM) domain (Figure 4C). This symmetrical interaction most probably corresponds to the dimeric short version of the fusion protein, whereas the intramolecular interaction (Figure 4A) could correspond to the monomeric form of the long version of the fusion protein (Figure 1).

The highly flexible activation loop of YabT(∆TM) is fully visible and displays an extended conformation stabilized by crystal packing interactions involving residues Lys173 and Arg176 and a neighboring bE8 molecule (Figure 4D). No residual electron density corresponding to phosphate groups could be observed close to the regulatory trans-autophosphorylation sites Thr171 and Thr172, suggesting that no significant autophosphorylation of the recombinant protein occurred during its production in *E. coli*.

### The Interaction With bE8 Stabilizes YabT(∆TM) in the Active Closed Conformation

**Figure 5:**
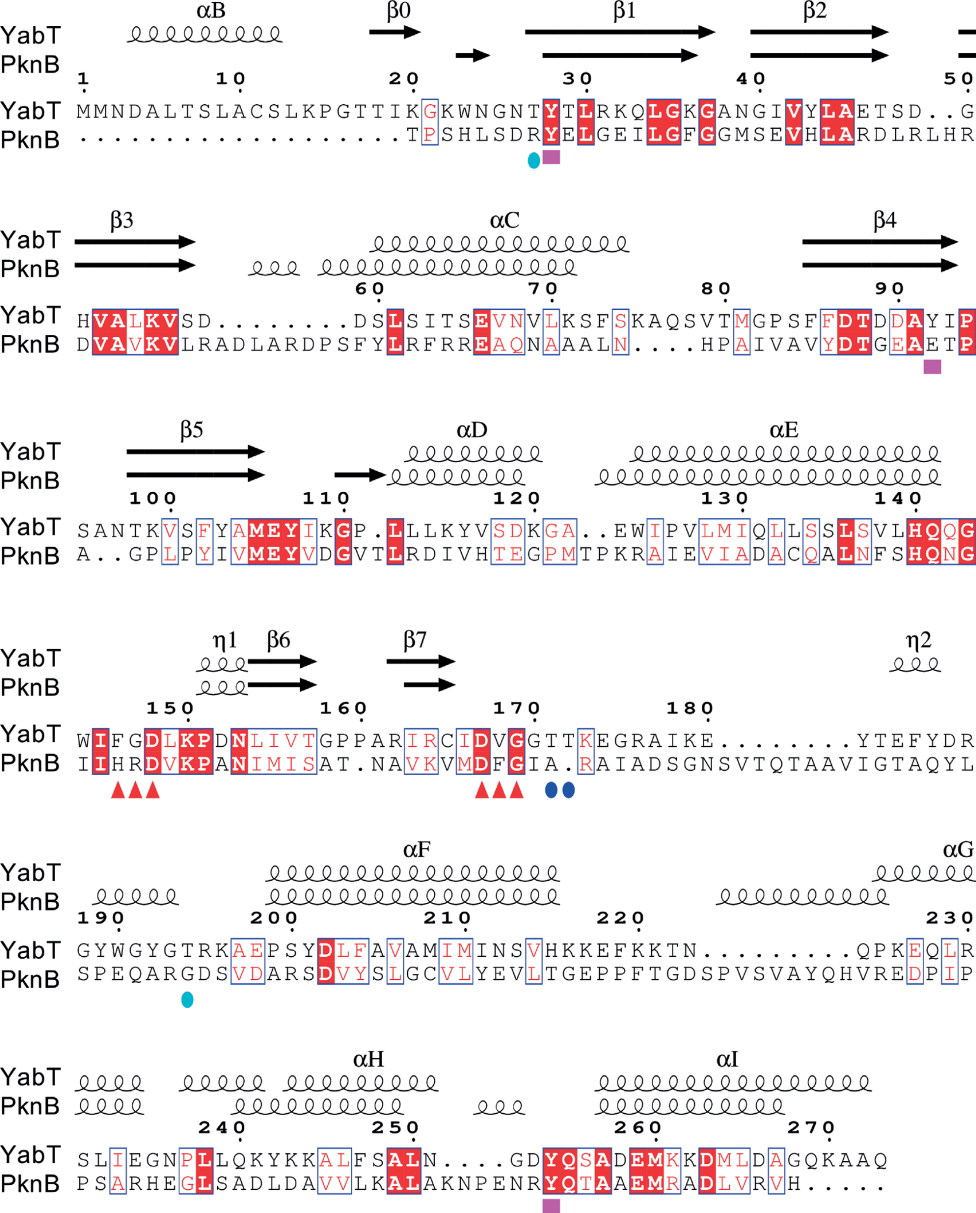
Sequence alignment between YabT and its closest structural homolog PknB from *Mycobacterium tuberculosis*. The secondary structure elements are shown at the top, using the PDB entry 2FUM for PknB. YabT residues discussed in the text are highlighted by logos at the bottom of the alignment: F^146^G^147^D^148^ and D^167^V^168^G^169^ motifs as red triangles, regulatory autophosphorylation sites T171 and T172 as blue spheres, and PtkA phosphorylation sites Y28, Y92, and Y254 as magenta squares. Figure prepared using ESPript (http://espript.ibcp.fr) (Robert and Gouet, 2014).

A search for structural relatives using the PDBeFold service^3^ (Krissinel and Henrick, 2004) revealed that the closest structural homolog of YabT is the cytosolic kinase domain of PknB from *M. tuberculosis* (PDB ID 2FUM) (Wehenkel et al., 2006). The latter displays only 21.4% sequence identity with YabT(∆TM) (Figure 5); however, their 3D structures showed strong similarity to each other with a rmsd of 2.1 Å over 210 aligned Cα atoms. YabT(∆TM) displays the structural characteristics of the active closed conformation of Hanks-type kinases. In particular, residues Glu66 from helix αC and Lys55 from strand β3 form the conserved salt bridge considered as a signature of the active kinase conformation (Kornev and Taylor, 2010). Lys55 also interacts with Asp167, which corresponds to the catalytic aspartate of the canonical Asp-Phe-Gly (DFG) motif (Figure 4D). The latter is degenerated into D^167^V^168^G^169^ in YabT (Figure 5). However, Val168 makes with helix αC characteristic hydrophobic interactions known to stabilize the closed conformation of the Hanks-type kinases (Nolen et al., 2004). The interaction with the αREP domain most probably stabilizes the kinase in this active and closed conformation, characteristic of the ATP-bound form of Hanks-type kinases.

### Influence of DNA Binding on the Structure of YabT

In the absence of bE8, ATP-binding and trans-autophosphorylation of YabT are enhanced in the presence of DNA (Bidnenko et al., 2013). We showed that the presence of the αREP domain in the bE8-YabT(∆TM) fusion protein does not impair the DNA-dependent phosphorylation process (Figure 3), despite stabilization of the closed conformation of the kinase. This suggests that the interaction between the two domains is dynamic, allowing YabT to switch to the open conformation in order to bind ATP in its interlobal cleft.

It has been shown that YabT is activated by DNA fragments with a minimal length of 15 bases (Bidnenko et al., 2013). The highly basic (pHi = 12.2) juxtamembrane segment responsible for DNA binding (residues 274–315), which is not visible in the crystal structure of the fusion protein, is also predicted as disordered in solution using the DisEMBL (Linding et al., 2003a) and GlobPlot (Linding et al., 2003b) tools (data not shown). This suggests that this small domain of 40 residues is unfolded in the absence of DNA. In order to determine the structure of this C-terminal flexible region in the presence of DNA, co-crystallization assays were performed using double-strand or single-strand oligonucleotides of various sizes. Unfortunately, no crystal of DNA-bound complex could be obtained, neither with YabT(∆TM) nor with the bE8-YabT(∆TM) fusion proteins. This is most probably due to the fact that YabT does not recognize a specific sequence of DNA, meaning that any fragments used for co-crystallization would bind heterogeneously.

**Figure 6:**
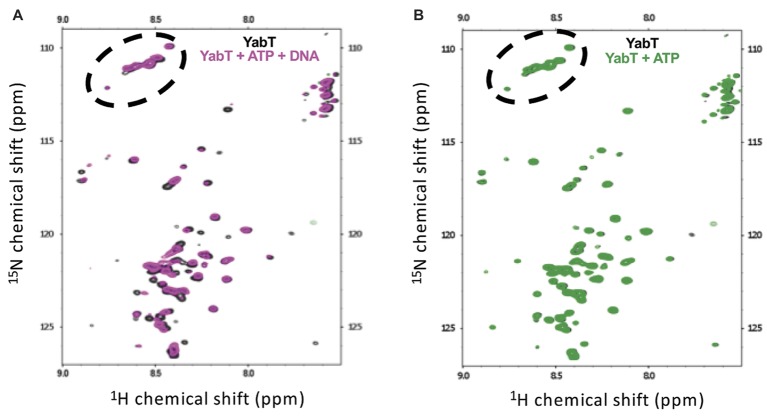
NMR analysis of YabT in the presence and absence of DNA. Superimposition of the Sofast ^1^H-^15^N HMQC NMR spectra of isotopically labeled 6His-YabT(∆TM) alone (in gray) **(A)**, after addition of ATP and DNA (in magenta), and **(B)**, after addition of ATP alone (in green). Each peak represents a bonded N-H pair, with its two coordinates corresponding to the chemical shifts of each of the H and N atoms. Only the region of the spectra corresponding to disordered residues is shown. The peaks corresponding to glycine residues are circled by dashed lines.

We further used NMR spectroscopy to test whether DNA binding could stabilize the flexible juxtamembrane region of YabT(∆TM). We recorded the sofast-HMQC spectrum of uniformly labeled YabT(∆TM) at 600MHz. The size of the protein is too large to observe all residues from the folded domain. In contrast, high-intensity signals likely corresponding to unfolded regions of the protein were identified. As shown in Figure 6, around 65 high-intensity peaks were observed in the region characteristic for unfolded tails. This corresponds to the expected number of signals from the unfolded C-terminal extension of YabT (43 residues including 7 prolines) and from the 6His-tag (29 residues including 8 glycines). Interestingly, addition of DNA to the ATP-bound protein induced variations of the intensity of the peaks as well as variation of the chemical shifts of a large proportion of these peaks (Figure 6A). The signals corresponding to glycines were not affected, consistent with the fact that the tag is not interacting with the DNA. As a control, we verified that addition of ATP, known to stabilize the active closed conformation of the catalytic domain of Hanks-type kinases, did not modify the high-intensity signals of the protein (Figure 6B). These results thus confirmed the hypothesis that the C-terminal extension of YabT(∆TM) is flexible and stabilized upon DNA binding.

## Discussion

Structural and functional studies of Hanks-type protein kinases revealed that the activating trans-autophosphorylation process entails a great deal of diversity in the underlying modes of dimerization (Lavoie et al., 2014). In particular, the closest structural homolog of YabT, that is, the mycobacterial kinase PknB, displays two modes of dimerization (Alber, 2009). In the presence of the bound ATP-competitive inhibitor KT5720, PknB forms an asymmetric dimer with the activation loop of one subunit facing the active site of the second subunit (PDB ID 3F69). A mutational analysis showed that this asymmetric face-to-face dimerization mode might play an essential role in the trans-autophosphorylation mechanism of the kinase (Mieczkowski et al., 2008). On the other hand, when bound to mitoxantrone, another ATP-competitive inhibitor, PknB displays an alternative dimerization mode characterized by a back-to-back interface (PDB ID 2FUM) (Wehenkel et al., 2006), which has been proposed to allosterically participate in the autophosphorylation process (Greenstein et al., 2007).

**Figure 7:**
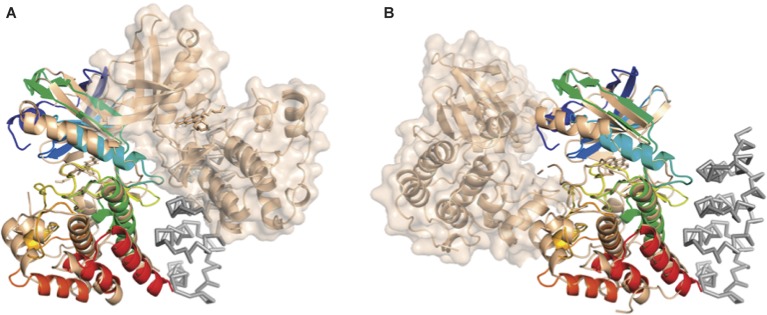
Comparison of the bE8-YabT(∆TM) structure with the two types of PknB dimerization modes. YabT(∆TM) is shown as cartoon colored in rainbow scheme. bE8 is shown as ribbon colored in grey. The PknB subunits are shown as cartoon colored in beige. The surface of the PknB subunit, which is not superimposed on YabT(∆TM), is shown in transparency. **(A)** Superimposition with the back-to-back dimer of PknB (PDB ID 2FUM). **(B)** Superimposition with the face-to-face PknB dimer (PDB ID 3F69).

Interestingly, superposition of the structure of the bE8-YabT(∆TM) fusion protein with both types of PknB dimers demonstrates that the bE8/YabT interaction is incompatible with the back-to-back dimer (Figure 7A) but not with the asymmetric face-to-face dimerization mode (Figure 7B). Unfortunately, the poor sequence identity between YabT and PknB did not allow us to identify contact residues of YabT in order to confirm this hypothesis.

**Figure 8:**
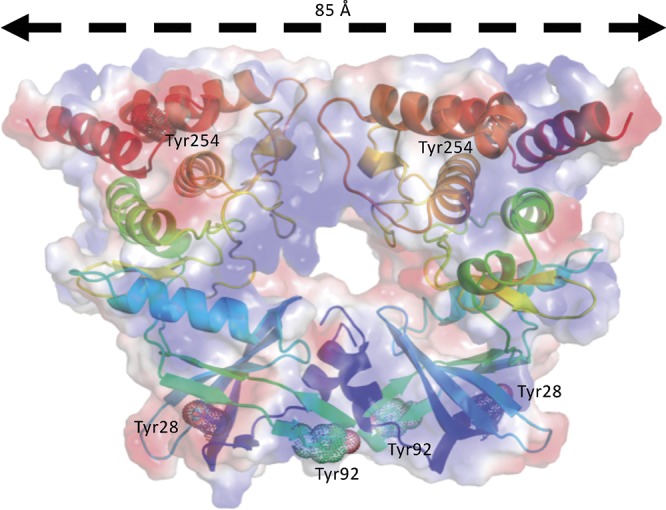
Model of a face-to-face YabT(∆TM) dimer. The model was built by superimposition of the structure of YabT(∆TM) on both subunits of the face-to-face dimer of PknB (PDB ID 3F69). The electrostatic surface of the dimer is shown in transparency with negatively and positively charged region colored in red and blue, respectively. Each polypeptide chain is shown as a cartoon trace colored in a rainbow scheme from blue to red. Tyrosine residues phosphorylated by the BY-kinase PtkA are highlighted by dots and labeled.

However, as reported above, autophosphorylation of the long form of the bE8-YabT(∆TM) fusion protein is activated upon DNA-binding (Figure 3). This suggests that this DNA-dependent activation mechanism could rely on the stabilization of a PknB-like active face-to-face YabT dimer (Figure 8). In this model, the C-termini of the two kinase subunits are located about 85 Å apart on each face of the YabT(∆TM) dimer, suggesting that the 40 juxtamembrane residues corresponding to the DNA-binding domain do not directly participate in dimer contacts. However, when YabT is anchored to the membrane at the septum of the sporulating bacteria (Bidnenko et al., 2013), binding of several YabT molecules to long stretches of DNA transported across the septum could favor intermolecular interactions compatible with trans-autophosphorylation and in particular the formation of face-to-face YabT dimers.

It has been suggested that tyrosine phosphorylation by the BY-kinase PtkA could represent another regulatory mechanism of YabT (Shi et al., 2014b). Two of the tyrosine residues that have been shown to be phosphorylated *in vitro* by PtkA, Tyr28 and Tyr254, are buried at the N-terminus and C-terminus of the protein, respectively. In turn, the third potential phosphorylation site, Tyr92, is exposed to the solvent at the surface of the protein, close to the potential contact region of the proposed face-to-face dimer (Figure 8). PtkA-dependent phosphorylation of Tyr92 could thus indeed further regulate YabT dimerization and trans-autophosphorylation.

In conclusion, our results shed some additional light on the mechanism of DNA-dependent activation of YabT. The kinase is known to be localized at the septum during spore formation (Bidnenko et al., 2013), where it is presumably activated by DNA entering the forespore. There, it phosphorylates RecA, which carries out chromosome quality control. A human kinase C-Abl also binds DNA and phosphorylates the RecA homologue Rad51, which is in turn involved in DNA repair (Shimizu et al., 2009). C-Abl and YabT are not activated by specific DNA sequences. C-Abl activity is known to be triggered by DNA damage, and we presently cannot exclude the possibility that YabT too could preferentially bind to damaged DNA. What can currently be inferred with a certain degree of confidence is that YabT monomers exist anchored to the membrane and are not localized before the onset of sporulation. At the onset of sporulation, YabT gets recruited at the septum, where it binds the passing DNA. According to the data presented herein, DNA binding supports the formation of face-to-face dimers, which in turn favors trans-autophosphorylation and activation of the kinase. YabT activation mechanism would thus be reminiscent of transmembrane protein kinase receptors, which are known to dimerize upon binding of the signal molecule to their external domain (Bocharov et al., 2017). For example, it has been proposed that binding of peptidoglycan fragments to the external PASTA domains of PknB would trigger dimerization and activation of the kinase (Barthe et al., 2010). In the case of YabT, the signal molecule would thus be the DNA transported through the septum. It would not only regulate phosphorylation of YabT substrates involved in genome maintenance, such as RecA, SSbA, and YabA, but also substrates involved in protein synthesis, such as AbrB and Ef-Tu, thus playing a possible additional regulatory role in the progressive metabolic quiescence of the forespore (Pereira et al., 2015). Further studies, both structural and *in vivo*, will be required to fully understand its activation and role during spore formation.

## Author Contributions

LS performed the EMSA experiments and the phosphorylation assays; AC produced, purified, and crystallized YabT(∆TM). J-LR produced, purified, and crystallized the fusion protein bE8-YabT(∆TM). NL purified the complex bE8/YabT(∆TM) and performed the SEC-MALS experiments. IS-G collected the diffraction data, FO performed and analyzed the NMR experiments, MV-L selected the bE8 binder specific of YabT(∆TM) and cloned the bE8-YabT(∆TM) fusion protein. AU and PM built the αREP library. IM supervised the molecular biology work, discussed the results, and corrected the manuscript. SN supervised the biochemistry work, solved, and analyzed the crystal structure of the bE8-YabT(∆TM) fusion protein and wrote the paper.

### Conflict of Interest Statement

The authors declare that the submitted work was not carried out in the presence of any personal, professional, or financial relationships that could potentially be construed as a conflict of interest.

## Footnotes

^1^ http://www.ebi.ac.uk/msd-srv/ssm/

^2^ http://www.ebi.ac.uk/pdbe/prot_int/pistart.html

^3^ http://www.ebi.ac.uk/msd-srv/ssm

## References

[ref1] Adams P. D. Afonine P. V. Bunkoczi G. Chen V. B. Davis I. W. Echols N.. (2010). PHENIX: a comprehensive Python-based system for macromolecular structure solution. Acta Crystallogr. D. Biol. Crystallogr. 66, 213–221. 10.1107/S0907444909052925, PMID: 20124702 PMC2815670

[ref2] Alber T. (2009). Signaling mechanisms of the *Mycobacterium tuberculosis* receptor Ser/Thr protein kinases. Curr. Opin. Struct. Biol. 19, 650–657. 10.1016/j.sbi.2009.10.017, PMID: 19914822 PMC2790423

[ref3] Barthe P. Mukamolova G. V. Roumestand C. Cohen-Gonsaud M. (2010). The structure of PknB extracellular PASTA domain from *Mycobacterium tuberculosis* suggests a ligand-dependent kinase activation. Structure 18, 606–615. 10.1016/j.str.2010.02.013, PMID: 20462494

[ref4] Bidnenko V. Shi L. Kobir A. Ventroux M. Pigeonneau N. Henry C.. (2013). *Bacillus subtilis* serine/threonine protein kinase YabT is involved in spore development via phosphorylation of a bacterial recombinase. Mol. Microbiol. 88, 921–935. 10.1111/mmi.12233, PMID: 23634894 PMC3708118

[ref5] Bocharov E. V. Sharonov G. V. Bocharova O. V. Pavlov K. V. (2017). Conformational transitions and interactions underlying the function of membrane embedded receptor protein kinases. Biochim. Biophys. Acta 1859, 1417–1429. 10.1016/j.bbamem.2017.01.025 28131853

[ref6] Brognard J. Hunter T. (2011). Protein kinase signaling networks in cancer. Curr. Opin. Genet. Dev. 21, 4–11. 10.1016/j.gde.2010.10.012, PMID: 21123047 PMC3038181

[ref7] Chevrel A. Mesneau A. Sanchez D. Celma L. Quevillon-Cheruel S. Cavagnino A. (2017). Alpha repeat proteins (alphaRep) as expression and crystallization helpers. J. Struct. Biol. 201, 88–99. 10.1016/j.jsb.2017.08.002 28823563

[ref8] Delano W. L. (2002). The PyMOL Molecular Graphics System. *on World Wide Web* http://www.pymol.org/. 10.1016/S1052-5149(03)00068-6, PMID: 11998251

[ref9] Derouiche A. Bidnenko V. Grenha R. Pigonneau N. Ventroux M. Franz-Wachtel M.. (2013). Interaction of bacterial fatty-acid-displaced regulators with DNA is interrupted by tyrosine phosphorylation in the helix-turn-helix domain. Nucleic Acids Res. 41, 9371–9381. 10.1093/nar/gkt709, PMID: 23939619 PMC3814354

[ref10] Derouiche A. Petranovic D. Macek B. Mijakovic I. (2017). *Bacillus subtilis* single-stranded DNA-binding protein SsbA is phosphorylated at threonine 38 by the serine/threonine kinase YabT. Period. Biol. 118, 399–404. 10.18054/pb.v118i4.4572

[ref11] Derouiche A. Shi L. Bidnenko V. Ventroux M. Pigonneau N. Franz-Wachtel M.. (2015). *Bacillus subtilis* SalA is a phosphorylation-dependent transcription regulator that represses scoC and activates the production of the exoprotease AprE. Mol. Microbiol. 97, 1195–1208. 10.1111/mmi.13098, PMID: 26094643

[ref12] Derouiche A. Shi L. Kalantari A. Mijakovic I. (2016). Substrate specificity of the *Bacillus subtilis* BY-kinase PtkA is controlled by alternative activators: TkmA and SalA. Front. Microbiol. 7, 1525. 10.3389/fmicb.2016.01525, PMID: 27725816 PMC5035731

[ref13] Deutscher J. Saier M. H. Jr (2005). Ser/Thr/Tyr protein phosphorylation in bacteria—for long time neglected, now well established. J. Mol. Microbiol. Biotechnol. 9, 125–131. 10.1159/000089641 16415586

[ref14] Emsley P. Cowtan K. (2004). Coot: model-building tools for molecular graphics. Acta Cryst D-Biol. Cryst. 60, 2126–2132. 10.1107/S0907444904019158, PMID: 15572765

[ref15] Foulquier E. Pompeo F. Freton C. Cordier B. Grangeasse C. Galinier A. (2014). PrkC-mediated phosphorylation of overexpressed YvcK protein regulates PBP1 protein localization in *Bacillus subtilis* mreB mutant cells. J. Biol. Chem. 289, 23662–23669. 10.1074/jbc.M114.562496, PMID: 25012659 PMC4156092

[ref16] Garcia Garcia T. Ventroux M. Derouiche A. Bidnenko V. Correia Santos S. Henry C. (2018). Phosphorylation of the *Bacillus subtilis* replication controller YabA plays a role in regulation of sporulation and biofilm formation. Front. Microbiol. 9, 486. 10.3389/fmicb.2018.00486 29619013 PMC5871692

[ref17] Gerwig J. Kiley T. B. Gunka K. Stanley-Wall N. Stulke J. (2014). The protein tyrosine kinases EpsB and PtkA differentially affect biofilm formation in *Bacillus subtilis*. Microbiology 160, 682–691. 10.1099/mic.0.074971-0, PMID: 24493247 PMC3973450

[ref18] Gouet P. Robert X. Courcelle E. (2003). ESPript/ENDscript: extracting and rendering sequence and 3D information from atomic structures of proteins. Nucleic Acids Res. 31, 3320–3323. 10.1093/nar/gkg556, PMID: 12824317 PMC168963

[ref19] Greenstein A. E. Echols N. Lombana T. N. King D. S. Alber T. (2007). Allosteric activation by dimerization of the PknD receptor Ser/Thr protein kinase from *Mycobacterium tuberculosis* J. Biol. Chem. 282, 11427–11435. 10.1074/jbc.M610193200, PMID: 17242402

[ref20] Guellouz A. Valerio-Lepiniec M. Urvoas A. Chevrel A. Graille M. Fourati-Kammoun Z.. (2013). Selection of specific protein binders for pre-defined targets from an optimized library of artificial helicoidal repeat proteins (alphaRep). PLoS One 8:e71512. 10.1371/journal.pone.0071512, PMID: 24014183 PMC3754942

[ref21] Hoch J. A. (2000). Two-component and phosphorelay signal transduction. Curr. Opin. Microbiol. 3, 165–170. 10.1016/S1369-5274(00)00070-9, PMID: 10745001

[ref22] Jaroszewski L. Rychlewski L. Li Z. Li W. Godzik A. (2005). FFAS03: a server for profile--profile sequence alignments. Nucleic Acids Res. 33, W284–W288. 10.1093/nar/gki418, PMID: 15980471 PMC1160179

[ref23] Jers C. Kobir A. Sondergaard E. O. Jensen P. R. Mijakovic I. (2011). *Bacillus subtilis* two-component system sensory kinase DegS is regulated by serine phosphorylation in its input domain. PLoS One 6:e14653. 10.1371/journal.pone.0014653, PMID: 21304896 PMC3033389

[ref24] Jers C. Soufi B. Grangeasse C. Deutscher J. Mijakovic I. (2008). Phosphoproteomics in bacteria: towards a systemic understanding of bacterial phosphorylation networks. Expert Rev Proteom. 5, 619–627. 10.1586/14789450.5.4.619 18761471

[ref25] Kabsch W. (2010). Xds. Acta Crystallogr D Biol Crystallogr. 66, 125–132. 10.1107/S0907444909047337, PMID: 20124692 PMC2815665

[ref26] Kobir A. Poncet S. Bidnenko V. Delumeau O. Jers C. Zouhir S.. (2014). Phosphorylation of *Bacillus subtilis* gene regulator AbrB modulates its DNA-binding properties. Mol. Microbiol. 92, 1129–1141. 10.1111/mmi.12617, PMID: 24731262

[ref27] Kornev A. P. Taylor S. S. (2010). Defining the conserved internal architecture of a protein kinase. Biochim. Biophys. Acta 1804, 440-444. 10.1016/j.bbapap.2009.10.017 19879387 PMC3435107

[ref28] Krissinel E. Henrick K. (2004). Secondary-structure matching (SSM), a new tool for fast protein structure alignment in three dimensions. Acta Crystallogr D Biol Crystallogr. 60, 2256–2268. Epub. 10.1107/S0907444904026460, PMID: 15572779

[ref29] Krissinel E. Henrick K. (2007). Inference of macromolecular assemblies from crystalline state. J. Mol. Biol. 372, 774–797. 10.1016/j.jmb.2007.05.022, PMID: 17681537

[ref30] Lavoie H. Li J. J. Thevakumaran N. Therrien M. Sicheri F. (2014). Dimerization-induced allostery in protein kinase regulation. Trends Biochem. Sci. 39, 475–486. 10.1016/j.tibs.2014.08.004, PMID: 25220378

[ref31] Leonard C. J. Aravind L. Koonin E. V. (1998). Novel families of putative protein kinases in bacteria and archaea: evolution of the “eukaryotic” protein kinase superfamily. Genome Res. 8, 1038–1047. 10.1101/gr.8.10.1038, PMID: 9799791

[ref32] Linding R. Jensen L. J. Diella F. Bork P. Gibson T. J. Russell R. B. (2003a). Protein disorder prediction: implications for structural proteomics. Structure 11, 1453–1459. 14604535 10.1016/j.str.2003.10.002

[ref33] Linding R. Russell R. B. Neduva V. Gibson T. J. (2003b). GlobPlot: exploring protein sequences for globularity and disorder. Nucleic Acids Res. 31, 3701–3708. 12824398 10.1093/nar/gkg519 PMC169197

[ref34] Lusetti S. L. Cox M. M. (2002). The bacterial RecA protein and the recombinational DNA repair of stalled replication forks. Annu. Rev. Biochem. 71, 71–100. 10.1146/annurev.biochem.71.083101.133940, PMID: 12045091

[ref35] Mader U. Antelmann H. Buder T. Dahl M. K. Hecker M. Homuth G. (2002). *Bacillus subtilis* functional genomics: genome-wide analysis of the DegS-DegU regulon by transcriptomics and proteomics. Mol. Gen. Genomics. 268, 455–467. 10.1007/s00438-002-0774-2, PMID: 12471443

[ref36] Mccoy A. J. Grosse-Kunstleve R. W. Adams P. D. Winn M. D. Storoni L. C. Read R. J. (2007). Phaser crystallographic software. J. Appl. Crystallogr. 40, 658–674. 10.1107/S0021889807021206, PMID: 19461840 PMC2483472

[ref37] Mieczkowski C. Iavarone A. T. Alber T. (2008). Auto-activation mechanism of the *Mycobacterium tuberculosis* PknB receptor Ser/Thr kinase. EMBO J. 27, 3186–3197. 10.1038/emboj.2008.236, PMID: 19008858 PMC2599879

[ref38] Mijakovic I. Poncet S. Boel G. Maze A. Gillet S. Jamet E.. (2003). Transmembrane modulator-dependent bacterial tyrosine kinase activates UDP-glucose dehydrogenases. EMBO J. 22, 4709–4718. 10.1093/emboj/cdg458, PMID: 12970183 PMC212725

[ref39] Nolen B. Taylor S. Ghosh G. (2004). Regulation of protein kinases; controlling activity through activation segment conformation. Mol. Cell 15, 661–675. 10.1016/j.molcel.2004.08.024, PMID: 15350212

[ref40] Olivares-Illana V. Meyer P. Bechet E. Gueguen-Chaignon V. Soulat D. Lazereg-Riquier S.. (2008). Structural basis for the regulation mechanism of the tyrosine kinase CapB from Staphylococcus aureus. PLoS Biol. 6:e143. 10.1371/journal.pbio.0060143, PMID: 18547145 PMC2422856

[ref41] Ortiz-Lombardia M. Pompeo F. Boitel B. Alzari P. M. (2003). Crystal structure of the catalytic domain of the PknB serine/threonine kinase from *Mycobacterium tuberculosis*. J. Biol. Chem. 278, 13094–13100. 10.1074/jbc.M300660200, PMID: 12551895

[ref42] Pereira S. F. Gonzalez R. L. Jr. Dworkin J. (2015). Protein synthesis during cellular quiescence is inhibited by phosphorylation of a translational elongation factor. Proc. Natl. Acad. Sci. U. S. A. 112, E3274–3281. 10.1073/pnas.1505297112, PMID: 26056311 PMC4485140

[ref43] Pompeo F. Byrne D. Mengin-Lecreulx D. Galinier A. (2018). Dual regulation of activity and intracellular localization of the PASTA kinase PrkC during *Bacillus subtilis* growth. Sci. Rep. 8, 1660. 10.1038/s41598-018-20145-2 29374241 PMC5786024

[ref44] Pompeo F. Foulquier E. Serrano B. Grangeasse C. Galinier A. (2015). Phosphorylation of the cell division protein GpsB regulates PrkC kinase activity through a negative feedback loop in *Bacillus subtilis*. Mol. Microbiol. 10.1111/mmi.13015, PMID: 25845974

[ref45] Postma P. W. Lengeler J. W. Jacobson G. R. (1993). Phosphoenolpyruvate:carbohydrate phosphotransferase systems of bacteria. Microbiol. Rev. 57, 543–594, PMID: 8246840 10.1128/mr.57.3.543-594.1993 PMC372926

[ref46] Robert X. Gouet P. (2014). Deciphering key features in protein structures with the new ENDscript server. Nucleic Acids Res. 42, W320–324. 10.1093/nar/gku316, PMID: 24753421 PMC4086106

[ref47] Shi L. Ji B. Y. Kolar-Znika L. Boskovic A. Jadeau F. Combet C. (2014a). Evolution of bacterial protein-tyrosine kinases and their relaxed specificity toward substrates. Genome Biol. Evol. 6, 800–817. 10.1093/gbe/evu056 24728941 PMC4007543

[ref48] Shi L. Pigeonneau N. Ravikumar V. Dobrinic P. Macek B. Franjevic D. (2014b). Cross-phosphorylation of bacterial serine/threonine and tyrosine protein kinases on key regulatory residues. Front. Microbiol. 5, 495. 10.3389/fmicb.2014.00495 25278935 PMC4166321

[ref49] Shi L. Pigeonneau N. Ventroux M. Derouiche A. Bidnenko V. Mijakovic I. (2014c). Protein-tyrosine phosphorylation interaction network in *Bacillus subtilis* reveals new substrates, kinase activators and kinase cross-talk. Front. Microbiol. 5, 538. 10.3389/fmicb.2014.00538 25374563 PMC4205851

[ref50] Shimizu H. Popova M. Fleury F. Kobayashi M. Hayashi N. Sakane I.. (2009). c-ABL tyrosine kinase stabilizes RAD51 chromatin association. Biochem. Biophys. Res. Commun. 382, 286–291. 10.1016/j.bbrc.2009.03.020, PMID: 19285032

[ref51] Shu J. C. Clarkson J. Yudkin M. D. (2004). Studies of SpoIIAB mutant proteins elucidate the mechanisms that regulate the developmental transcription factor sigmaF in *Bacillus subtilis*. Biochem. J. 384, 169–178. 10.1042/BJ20040923, PMID: 15294015 PMC1134100

[ref52] Stancik I. A. Sestak M. S. Ji B. Axelson-Fisk M. Franjevic D. Jers C.. (2018). Serine/threonine protein kinases from bacteria, archaea and eukarya share a common evolutionary origin deeply rooted in the tree of life. J. Mol. Biol. 430, 27–32. 10.1016/j.jmb.2017.11.004, PMID: 29138003

[ref53] Urvoas A. Guellouz A. Valerio-Lepiniec M. Graille M. Durand D. Desravines D. C.. (2010). Design, production and molecular structure of a new family of artificial alpha-helicoidal repeat proteins (alphaRep) based on thermostable HEAT-like repeats. J. Mol. Biol. 404, 307–327. 10.1016/j.jmb.2010.09.048, PMID: 20887736

[ref54] Valerio-Lepiniec M. Urvoas A. Chevrel A. Guellouz A. Ferrandez Y. Mesneau A.. (2015). The alphaRep artificial repeat protein scaffold: a new tool for crystallization and live cell applications. Biochem. Soc. Trans. 43, 819–824. 10.1042/BST20150075, PMID: 26517888

[ref55] Van Schaik W. Tempelaars M. H. Zwietering M. H. De Vos W. M. Abee T. (2005). Analysis of the role of RsbV, RsbW, and RsbY in regulating {sigma}B activity in *Bacillus cereus*. J. Bacteriol. 187, 5846–5851. 10.1128/JB.187.16.5846-5851.2005, PMID: 16077134 PMC1196065

[ref56] Wehenkel A. Bellinzoni M. Grana M. Duran R. Villarino A. Fernandez P. (2008). Mycobacterial Ser/Thr protein kinases and phosphatases: physiological roles and therapeutic potential. Biochim. Biophys. Acta 1784, 193–202. 10.1016/j.bbapap.2007.08.006 17869195

[ref57] Wehenkel A. Fernandez P. Bellinzoni M. Catherinot V. Barilone N. Labesse G.. (2006). The structure of PknB in complex with mitoxantrone, an ATP-competitive inhibitor, suggests a mode of protein kinase regulation in mycobacteria. FEBS Lett. 580, 3018–3022. 10.1016/j.febslet.2006.04.046, PMID: 16674948

[ref58] Yeats C. Finn R. D. Bateman A. (2002). The PASTA domain: a beta-lactam-binding domain. Trends Biochem. Sci. 27, 438. 10.1016/S0968-0004(02)02164-3, PMID: 12217513

[ref59] Young T. A. Delagoutte B. Endrizzi J. A. Falick A. M. Alber T. (2003). Structure of *Mycobacterium tuberculosis* PknB supports a universal activation mechanism for Ser/Thr protein kinases. Nat. Struct. Biol. 10, 168–174. 10.1038/nsb897, PMID: 12548283

